# Shoulder Calcific Tendinitis Presenting as Septic Arthritis: A Case Report

**DOI:** 10.7759/cureus.95595

**Published:** 2025-10-28

**Authors:** Ibrahim M Aljumaan, Abdullah S Alzahrani, Hayat A Khan, Asail Ahmed A Alammar, Anwar Alnakhli

**Affiliations:** 1 Orthopedic Surgery, King Fahad Military Medical Complex, Dhahran, SAU; 2 Orthopaedic Surgery, Dr. Sulaiman Al Habib Hospital, Riyadh, SAU; 3 General Medicine, King Fahad Medical City, Second Health Cluster, Riyadh, SAU

**Keywords:** acute shoulder pain, bursitis, calcific tendonitis, limited shoulder motion, septic arthritis

## Abstract

Calcific tendinitis of the shoulder is a common condition in which calcium hydroxyapatite crystals form within the rotator cuff tendons, most often the supraspinatus. As a sequela of this condition, long-term discomfort may develop with intermittent acute flares. Symptoms can also arise suddenly, causing intense pain and limited shoulder motion. These effects are often reflected in patient-reported outcome measures, for example, overall limitations in upper-extremity function and overhead activity. During flares, symptoms can mimic septic arthritis. This occurs on clinical exams and imaging. Accurate diagnosis is therefore difficult. Misdiagnosis may lead to unnecessary procedures or inappropriate treatment. Careful assessment with ultrasound and MRI helps distinguish calcific tendinopathy from infection. Recognizing typical features and choosing the right imaging are essential for optimal care. A 42-year-old man with a year of right-shoulder pain had a sudden flare with fever and chills, raising concern for septic arthritis. Despite normal inflammatory markers, empiric IV antibiotics were started; MRI then showed supraspinatus calcific deposits and subacromial-subdeltoid bursitis without effusion or infection. Antibiotics were stopped, and the diagnosis was revised to acute calcific tendinitis. He underwent arthroscopic debridement with tendon repair, followed by physiotherapy, with marked symptomatic and functional improvement. Acute calcific tendinopathy (ACT) of the shoulder often mimics septic arthritis. Abrupt pain, restricted motion, and occasional marker elevation, sometimes fever with crystal rupture, can prompt misdiagnosis and unwarranted antibiotics or surgery. Diagnosis rests on exam plus imaging: radiographs/ultrasound show calcifications and MRI helps exclude infection. In our case, early imaging and multidisciplinary review prevented unnecessary antimicrobials and enabled timely arthroscopy.

## Introduction

Calcific tendonitis is a common, non-traumatic cause of shoulder pain characterized by the buildup of calcium hydroxyapatite crystals in the tendons of the rotator cuff, most often the supraspinatus. The etiology remains unclear, although localized hypoxia, mechanical overload, and metabolic conditions are believed to contribute to crystal formation. While typically self-limiting, the condition can present atypically with acute flares that pose diagnostic challenges [1-2].

These acute episodes may present similarly to other serious conditions, such as septic arthritis, subacromial bursitis, or rotator cuff tears, making diagnosis more difficult. Such flares are believed to be triggered by rupture of calcium deposits into nearby tissues, causing an intense inflammatory reaction. Although symptoms like low-grade fever are uncommon, they may lead clinicians to suspect infection [3].

On physical examination, signs such as warmth and erythema around the shoulder are usually mild or absent. Blood tests, including white cell count and inflammatory markers, may be normal or only slightly elevated, and further investigation is warranted, including X-rays, ultrasound, or magnetic resonance imaging (MRI). These studies help confirm the presence of calcium deposits and rule out more serious infection [4-8].

Management strategies range from conservative treatments, such as nonsteroidal anti-inflammatory drugs (NSAIDs), physical therapy, and extracorporeal shockwave therapy, to more invasive interventions, including ultrasound-guided lavage and arthroscopic debridement, particularly in cases resistant to initial therapy [9].

## Case presentation

A 42-year-old male, previously healthy, presented with a one-year history of intermittent right shoulder pain, gradually worsening with activity. The pain was localized to the deltoid region and radiated to the right arm. He reported difficulty raising his arm above shoulder level. There was no history of trauma.

He had been managed conservatively over the past year with NSAIDs, physiotherapy, and a corticosteroid injection; however, his symptoms persisted.

On clinical examination, the patient demonstrated tenderness and stiffness along the shoulder joint line. Several provocative tests were positive, including O’Brien’s test, Jobe’s test, Neer sign, empty-can test, Speed’s test, lift-off test, and a painful arc. These findings were suggestive of a possible rotator cuff tear and warranted further imaging evaluation to confirm the diagnosis and guide management.

Acute flare of shoulder pain

The patient was scheduled for admission; one week prior, he developed an acute exacerbation with fever (38.9 °C), chills, and severe worsening of shoulder pain, prompting an emergency department visit.

Vital signs are as follows: temperature, 38.9°C; heart rate, 102 bpm; blood pressure, 125/76 mmHg; and respiratory rate, 18 breaths per minute.

Physical findings revealed right shoulder localized tenderness and markedly reduced range of motion. No erythema or swelling was noted. Neurovascular status was intact. Systemic examination was unremarkable.

Initial laboratory tests showed a white blood cell count of 10.6 × 10⁹/L (normal: 4.0-11.0 × 10⁹/L), an erythrocyte sedimentation rate (ESR) of 28 mm/hr (normal: < 20 mm/hr), and a C-reactive protein (CRP) level of 9 mg/L (normal: < 5 mg/L), indicating mild elevation across inflammatory markers. While these findings were not diagnostic, they raised concern in the context of the patient’s clinical presentation. As a precautionary measure, the patient was started empirically on intravenous antibiotics. Blood cultures were obtained, and an MRI was arranged to further evaluate the suspected joint infection.

The synovial fluid analysis demonstrated characteristics that argued against septic arthritis, including a clear appearance, a low white blood cell count (<2,000 cells/µL), a neutrophil predominance of less than 25%, and normal glucose and protein levels. In addition, both Gram stain and culture were negative, further excluding the likelihood of a septic joint.

Shoulder MRI

**Figure 1:**
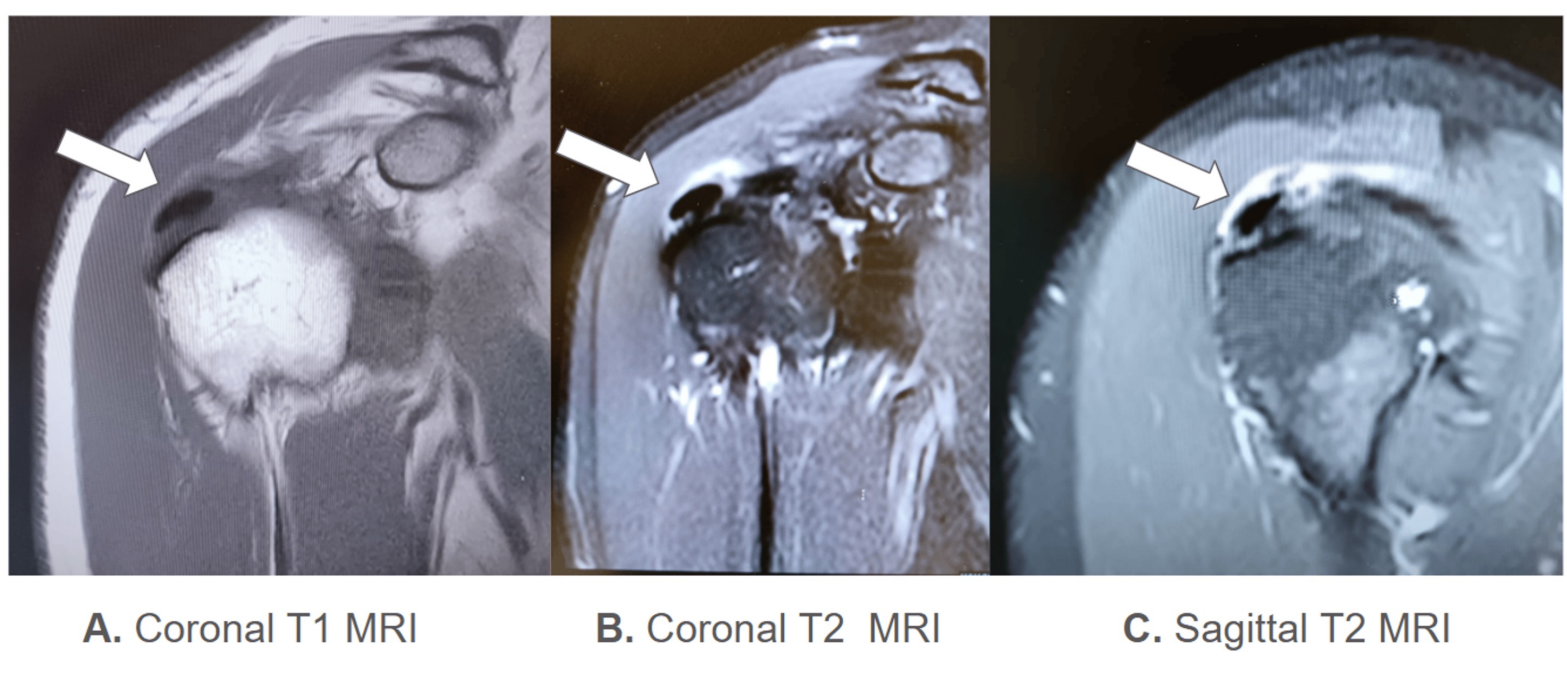
MRI showing calcification within supraspinatus tendon insertion

Multiple oblong low-signal lesions within the distal supraspinatus tendon fibers were observed, consistent with calcific deposits. Moderate subacromial-subdeltoid bursitis and mild AC joint osteoarthritis were also found. Infraspinatus, subscapularis, and biceps tendons were unremarkable. There was no joint effusion or bone involvement and no labral or marrow pathology (Figure 1).

Revised management

IV antibiotics were discontinued after the infection was ruled out. The patient was referred to orthopedic surgery for definitive management. He was continued on NSAIDs and analgesics and advised to rest the shoulder while performing a gentle range of motion exercises. Arthroscopic debridement was planned to remove the calcific deposits.

Impression

The systemic symptoms were presumed to be reactive or inflammatory in nature rather than infectious. Imaging and arthroscopic findings confirmed calcific tendonitis of the supraspinatus tendon with mild subacromial-subdeltoid bursitis, without features suggestive of septic arthritis. The final diagnosis was an acute flare of shoulder calcific tendonitis with subacromial-subdeltoid bursitis.

Surgical intervention

Shoulder arthroscopy was performed, which confirmed calcific tendonitis of the supraspinatus tendon. Debridement of the calcified material was carried out, followed by tendon repair using FiberWire sutures. A local steroid injection (Depo-Medrol) was administered. The arm was placed in a sling postoperatively, with instructions to avoid active movement initially. The patient was then referred to physiotherapy to begin a progressive rehabilitation program.

Outcome and follow-up

The patient tolerated the procedure well and was started on a structured physiotherapy program. At follow-up, he reported significant pain relief, improved range of motion, and no recurrence of fever or other systemic symptoms.

## Discussion

Rarity of acute/systemic presentation

Acute calcific tendonitis may resemble other serious shoulder pathologies, such as septic arthritis, cervical radiculopathy, subacromial bursitis, or even a rotator cuff tear. These acute flares are believed to result from rupture of calcium deposits into adjacent soft tissues, triggering a strong localized inflammatory reaction [3-5].

Diagnostic pitfalls and role of imaging

The diagnostic overlap between acute calcific tendonitis and septic arthritis makes early and accurate diagnosis essential. While elevated ESR, CRP, and leukocytosis may suggest infection, their presence is not definitive, and their absence does not rule it out. Thus, diagnosis should integrate clinical examination with appropriate imaging [6].

Plain radiography remains a first-line tool due to its sensitivity in detecting calcific deposits. Ultrasound offers a cost-effective, point-of-care modality that can visualize echogenic foci with posterior acoustic shadowing, often guiding needling or lavage procedures [7]. MRI provides high-resolution detail of soft tissue and osseous structures, helping differentiate between infection, bursitis, and tendon rupture [8].

In the case presented, the patient showed signs that increased the likelihood of septic arthritis, including systemic symptoms and raised inflammatory markers. However, imaging studies, including X-rays and MRI, along with a sterile joint aspiration, pointed to a diagnosis of acute calcific tendonitis.

Therapeutic options and indications for surgery

Calcific tendonitis can be managed in various ways, ranging from conservative treatments to surgery. Most cases start with conservative options like anti-inflammatory medications (NSAIDs), steroid injections, and physical therapy. In some situations, less invasive procedures such as ultrasound-guided lavage or barbotage would also be effective [3-5].

In our patient, initial conservative treatment failed to relieve symptoms, and the severity of the acute episode warranted surgical intervention. The successful arthroscopic debridement not only provided immediate pain relief and restored function but also allowed for direct visualization and repair of the affected tendon. This underscores the value of individualized, stepwise escalation of care and the confidence in the successful outcomes of surgical intervention.

Avoiding unnecessary antibiotic use

One critical implication of our case is the importance of distinguishing inflammatory from infectious processes to prevent unnecessary antibiotic use. Empirical antibiotic therapy, although often initiated in suspected septic arthritis, carries risks including resistance, side effects, and healthcare costs. Utilization of imaging early in the diagnostic process, ideally in collaboration with expert musculoskeletal radiologists, can mitigate these risks. While decision rules such as the Kocher criteria assist in pediatric hip infections, similar evidence-based tools are needed for shoulder conditions [3]. 

Clinical implications

Our case provides valuable insights into managing acute calcific tendinitis. Although generally mild, it can occasionally present with fever and inflammation that resemble infection, complicating diagnosis. Early imaging, particularly X-rays and ultrasound, is important to help rule out more serious concerns. Further discussion with the imaging team is often necessary to reach an accurate diagnosis. If conservative treatments do not provide relief, arthroscopy can both confirm the diagnosis and treat the problem effectively.

Table 1 shows the differentiation between shoulder calcific tendonitis and septic arthritis.

**Table 1:** Differentiation between shoulder calcific tendonitis and septic arthritis

Category	Calcific tendonitis [1, 3-5, 7-12]	Septic arthritis [8-10]
Onset	Present with either a gradual onset or a sudden acute flare, which can closely resemble septic arthritis in its early phase, both clinically and radiographically	Sudden and rapid onset of pain and dysfunction, commonly with systemic signs
Pain	Severe localized shoulder pain, especially during acute flare, worsens with movement	Severe and diffuse pain associated with joint inflammation
Systemic symptoms	Usually absent in calcific tendinopathy; however, low-grade fever may occur during acute flares, leading to diagnostic confusion with infection	Common: high fever, chills, and systemic inflammatory response
Range of motion	Painful active motion, but passive range may be partly preserved.	Marked limitation of both active and passive range due to effusion and pain
Tenderness	Focal tenderness over the rotator cuff (especially supraspinatus) insertion	Generalized joint tenderness, especially on palpation and passive motion
Erythema and warmth	Local erythema and warmth in acute calcific tendinopathy are typically absent or mild, which may help differentiate it from true septic arthritis.	Often present with visible erythema, warmth, and swelling
Leukocytosis	In the acute phase of calcific tendinopathy, leukocytosis is often absent or only mildly elevated, which may complicate differentiation from joint infection.	Markedly elevated white blood cell count
ESR / CRP	Mild to moderate elevation during acute flare	Typically very high inflammatory markers
Shoulder aspiration	Usually not required; aspirate is sterile if performed.	Essential for diagnosis; shows purulent fluid with positive culture
X-ray findings	Shows calcific deposits in the rotator cuff, mainly the supraspinatus	May appear normal early; late stages show joint destruction
Ultrasound	Detects hyperechoic calcifications and helps guide needling	Shows joint effusion and synovial thickening
MRI	Edema around tendons with calcific foci may mimic infection.	Effusion, synovial enhancement, and marrow edema suggest infection.
Definitive diagnosis	Based on imaging: X-ray, ultrasound, and MRI findings	Confirmed via joint aspiration and positive culture
Treatment	Conservative (NSAIDs, physiotherapy, shockwave therapy) or intervention (US-guided needling, arthroscopy)	Urgent IV antibiotics and surgical drainage to prevent joint destruction
Prognosis	Excellent with conservative or minimally invasive treatment; self-limited course	Guarded prognosis; delayed treatment may result in permanent damage.

## Conclusions

This case illustrates how an acute flare of calcific tendonitis can closely resemble septic arthritis. Early imaging, particularly MRI, is crucial to differentiate these entities and prevent unnecessary antibiotic use. When conservative measures fail or symptoms persist, arthroscopic debridement is an effective therapeutic option.

We recommend maintaining a high index of suspicion for calcific tendonitis in patients presenting with acute shoulder pain, even when systemic symptoms (e.g., fever) are present, prioritizing imaging before initiating antimicrobial therapy, and actively mitigating anchoring bias during clinical decision-making. Further development and validation of diagnostic algorithms or clinical scoring systems for adult shoulder infections may enhance future management.
